# Microencapsulation of engineered bacteria towards whole-cell-based environmental biosensing

**DOI:** 10.3389/fbioe.2026.1851287

**Published:** 2026-06-30

**Authors:** Luana Cuvillier, Guillaume Nonglaton

**Affiliations:** Univ. Grenoble Alpes, CEA-Leti, Grenoble, France

**Keywords:** alginate-poly-L-lysine microcapsules, cell confinement, diffusion modelling, layer-by-layer encapsulation, long-term viability, whole-cell biosensor

## Abstract

**Introduction:**

Legitimate concerns about the quality of environmental waters are on the rise. As a consequence, efforts are being made by both the scientific community and policymakers to develop proficient methods for the monitoring and detection of pollutants of emerging concerns (PECs). In line with the European Union’s “Zero Pollution” action plan, there is a growing need to develop real-time, multiplexed, on-site monitoring systems. The study explores the encapsulation of engineered *Pseudomonas putida* cells designed to produce a luminescent response upon exposure to specific analytes. The aim was to develop a suitable, miniaturized, biocompatible, safe-and-sustainable-by-design, sensing element for future integration with optical-electrochemical transduction systems.

**Methods:**

Genetically modified *P. putida* cells were encapsulated in sub-200 µm alginate microcapsules using a layer-by-layer method with poly-L-lysine (PLL) to avoid bacterial escape. Capsules morphology and structure were characterized using Laser Scanning Confocal and cryo-Scanning Electron microscopies. Effects of cell load, storage temperature, and storage medium were evaluated through encapsulated cells’ fluorescent response to induction. Long-term fluorescent activity was evaluated over a 2-month period. Response-time of the encapsulated cells was investigated using both experimental and diffusion modelling approaches.

**Results:**

Encapsulation strategy using alginate-PLL capsules revealed a good mechanical stability and resistance to saline water, with minimal cell leakage. About 75% of encapsulated cells remained viable after 45 days, and fluorescence increase upon induction was still observed after 2 months of storage at room temperature without nutrient supply. These findings suggest that a subpopulation of cells entered a dormant or low-metabolic state, enabling long-term sensing under non-ideal conditions. Higher cell loads correlated with stronger responses, likely due to an increased fraction of metabolically reactivable cells. Encapsulated cells were also reusable, showing measurable sensing response for five sensing cycles within 2 weeks.

**Discussion:**

Results demonstrate that response dynamics are primarily governed by reduced metabolic activity and limited oxygen access, rather than by analytes diffusion through the hydrogel matrix.

## 1 Introduction

Pollution is the largest environmental cause of premature deaths, reaching 9 million deaths worldwide in 2015, *i.e.* 16 % of total deaths (Fuller et al., 2022). Population growth, urbanization, industrialization, and the unsustainable use of natural resources have contributed to increased environmental pollution. More recently, substances labelled as pollutants of emerging concerns (PECs) are increasingly contaminating our natural water sources (Directive, 2020). Such pollutants include endocrine-disruptive chemicals (EDCs), per- and polyfluoroalkyl substances (PFAS), heavy metals, microplastics, pharmaceuticals, and agricultural or industrial wastes containing nitrogen or phosphorus. This has led to biodiversity loss and global ecosystem degradation, posing multiple risks to both animal and human health (Fuller et al., 2022). These are of particular concern as they can disrupt metabolic functions, reproduction or overstimulate microbial blooms (Yaakob et al., 2021; Kidd et al., 2007; Pezeshki et al., 2025).

To address this issue, action plans aimed at preventing, monitoring, reporting and remediating water and soil pollution have recently been launched by different authorities (European Commission, 2021). Whenever prevention at the source is not yet possible, pollution tracking solutions should be promoted as complementary strategies. This work therefore aims to contribute to the development of an on-site microbial whole-cell-based bimodal biosensor, combining optical and electrochemical transducers, for the *in situ* monitoring of PFAS, EDCs, microcystins and soil nutrients.

A key challenge is the lack of effective digital monitoring tools providing real-time sensing of harmful chemicals. Surveillance of these substances requires detection techniques that can operate on-site, provide results within hours, and reliably detect trace concentrations. Currently, reliable real-time on-site measurement remains limited for most of the above-cited pollutants. Existing methods rely on laboratory-based approaches that are costly, time-consuming and logistically demanding, mainly gas or liquid chromatography or ELISA (Nahar et al., 2023; Sosa-Ferrera et al., 2013; Moshoeshoe and Veronica, 2018). In addition to extensive sample preparation (*i.e.*, extraction, pre-concentration), these technologies require expensive and bulky analytical instruments, preventing large-scale, real-time environmental monitoring. For these reasons, there is an increased interest in the development of field-deployable sensors for pollutants detection (Khitous et al., 2025; Zhu et al., 2024).

Microbial sensors based on whole-cells as sensing elements represent a promising alternative, and the field has seen significant progress over the past decade (Thouand, 2022; Belkin and Wang, 2021) Microorganisms can be engineered to produce measurable outputs in response to specific analytes, thereby functioning as whole-cells-based biosensors (WCB) (Bergman et al., 2025; McKenny and Riglar, 2026). WCB sensors use living microbes as microfactories, able to recognize target analytes, process information, and produce a standardized, detectable signal that can be transmitted to suitable transducers. Such microbial devices have been used for diverse applications, including environmental monitoring (Buffi et al., 2011; Gui et al., 2017; Zhou et al., 2026; Li et al., 2024), monitoring of substrates and metabolites in bioprocess industries (Moya-Ramírez et al., 2022), and disease diagnostics (Aghlara-Fotovat et al., 2023). Recent advances in synthetic biology enable precise tuning of cellular response to specific contaminants with output types including optical signals (*i.e.*, chemiluminescence, fluorescence, absorbance), or electrical signals (*i.e.*, amperometric, conductometric, potentiometric). (Hicks et al., 2020; Atkinson et al., 2022; Hernández-Sancho et al., 2024).

In this work, a *Pseudomonas putida* strain was genetically modified to produce an optically active molecule, namely, superfolder Green Fluorescent Protein (sfGFP). Rhamnose was used as an inducer as a proof-of-concept prior to developing a functional water pollutant sensor. This work represents a preliminary step toward future studies involving a real pollutant rather than rhamnose.

For practical deployment, these bioengineered cells are incorporated into a miniaturized, automated, closed device connected to optical and electrochemical transducers. Immobilization of microbial biosensors within the device chamber is essential to ensure both cell preservation and confinement, consistent with a safe and sustainable by design (SSbD) approach. Indeed, effective immobilization not only improves cell lifespan, reducing the need to change the sensor (Aghlara-Fotovat et al., 2023), but also prevents the escape of genetically modified microorganisms during on-site monitoring, thus improving biosafety.

Cell encapsulation is widely used in biotechnology and medicine, including environmental remediation, bioproduction, probiotic delivery, drug release, and tissue regeneration (Anselmo et al., 2016; Perullini et al., 2015; Mehrotra et al., 2020). Several encapsulation or immobilization strategies have been documented and studied, including sol-gel (Nassif et al., 2003; Wang et al., 2015; Ghach et al., 2014), or agar-based systems (Buffi et al., 2011). Integration into biomaterial carriers is one of the most widespread methods, providing a protective and semi-controllable environment to support the viability of encapsulated cells (Aghlara-Fotovat et al., 2023; Gasperini et al., 2014). In addition to common encapsulation constraints (*i.e.*, cell viability and biocompatibility), whole-cell-based sensor-specific limitations must also be considered, particularly adequate diffusion of target pollutants and output molecules within the chamber, as well as the absence of signal transmission attenuation (Erdogan et al., 2025). Alginate has attracted significant attention due to its low cost, biocompatibility, and ability to form ionically crosslinked hydrogels. These hydrogels create semi-permeable matrices that allow the diffusion of small molecules while offering favorable physicochemical properties such as water insolubility and optical transparency. In particular, the layer-by-layer (LbL) strategy, consisting of the sequential deposition of thin films in a fast, economical, and controllable manner, results in an inner hydrogel core coated by an additional material layer and appears to reduce bacterial escape (Moya-Ramírez et al., 2022; Bruyant and Coradin, 2007). Furthermore, microencapsulation in the form of spherical beads provides several advantages compared with two-dimensional film structures fabricated by spin coating or drop-casting, notably a higher surface area and the ability to introduce capsules into a pre-assembled chamber through a Luer filling port.

To demonstrate this potential, this paper shows how *P. putida* cells engineered to produce a green fluorescent protein (sfGFP) in response to rhamnose, can be encapsulated in approximately 180 μm diameter alginate beads. These beads are intended to be integrated into miniaturized dual optical-electrochemical transducer chambers for water pollutants monitoring.

The objectives of this work are to develop and characterize a bacterial cell encapsulation process compatible with the constraints of immobilizing whole-cell sensors in compact bimodal transducers including size, prevention of cell escape, signal transmission, and transducer compatibility (Supplementary Figure S1). In parallel, this work aims to investigate and optimize both the response time and the longevity of encapsulated cell sensing activity, with particular emphasis on long-term viability and inducible functionality under confined and nutrient-limited conditions. Particular attention is devoted to understanding how the “living sensor” component maintains functionality over time within a confined, nutrient-limited device environment.

## 2 Materials and methods

### 2.1 Chemicals and materials

Alginic acid sodium salt from brown algae (BioReagent, quality level 100), poly-L-lysine hydrobromide powder (molecular weight 30,000–70,000), anhydrous granular calcium chloride (ReagentPlus, ≥93%), agar (quality level 100), LB broth (Lennox), phosphate-buffered saline (PBS) (liquid, 10X), sodium chloride, trehalose, yeast extract, and soy peptone were purchased from Sigma-Aldrich. Monohydrated L-(+)-rhamnose (99%), was purchased from Thermo Scientific Chemicals. Kanamycin monosulfate was obtained from Euromedex. All reagents were used without further purification.

### 2.2 Bacterial strains and growth conditions

The strain used was *P. putida EM42,* with specific sensing mutations. It was kindly provided by the Bioprocess Engineering group of Wageningen University (Netherlands). The strain EM42 is a derivative of the *P. putida* wild type strain KT2440, with a deletion of the flagella-encoding gene, resulting in the removal of the entire flagellar machinery. It tolerates endogenous oxidative stress and demonstrates better survival in the stationary phase (Amendola et al., 2024; Martínez-García and Pablo, 2014). *P. putida* EM42 was further modified by insertion of plasmids to create two model sensors. Both final strains carry a plasmid based on the pSEVA23b backbone, encoding a kanamycin selection marker and the rhamnose-inducible two-component system *rhaS/rhaR* for controlled gene expression. Additionally, the plasmid either contains a rhamnose-inducible *superfolder GFP* (*sfGFP*) gene (pSEVA23b_pRham_sfGFP), for a final strain referred to as PPGFP, or a rhamnose-inducible *sfGFP* gene fused to the SsrA degradation tag (pSEVA23b_pRham_sfGFP_SsrA) to increase the rate of protein turnover (Martínez-García et al., 2020), referred to as PPGFP-SsrA. From −80 °C, strains were plated on self-prepared LB-agar plates (20 g·L^−1^ LB broth and 15 g·L^−1^ agar) containing 50 μg mL^−1^ kanamycin for GFP expressing strains. Plated strains were incubated overnight at 30 °C and kept at 4 °C until use, for no longer than 7 days after initial plating. From these plates, under sterile conditions, a single colony was inoculated in 100 mL sterile LB broth with 50 μg mL^−1^ kanamycin in 500 mL sterile Erlenmeyer flasks. Flasks were sealed with BREATHseal™ membranes (Greiner Bio-One Intl, Austria) and incubated at 30 °C with agitation at 200 rpm for 16 h. Cultures were centrifuged (4,000 rpm, 15 min, 4 °C) and washed once with PBS (1X) and twice with Milli-Q water. Cells were then resuspended in Milli-Q water to the desired concentration (OD_600_) and stored at 4 °C until use.

### 2.3 Size optimization and coating of alginate micro-capsules

Monodispersed calcium alginate microbeads (Alg) were produced using a protocol developed by Badalan et al. (2022) adapted by Bassut *et al.* and further modified in this work (Bassut et al., 2025). 50 mL falcon tubes were filled with 100 mM (11 g·L^−1^) CaCl_2_ crosslinking solution. The tube lids were modified to hold a 5 mL syringe (Luer-Lok™ Tip, BD), previously cut at the 1 mL mark. CellInk® blunt needles (straight, green, 34G, 6.35 mm, ⌀ 0.06 mm inner diameter) were connected to the syringe through Luer-Lock connection. The distance (h) between the needle tip and the crosslinking bath was adjusted by varying the volume of CaCl_2_ solution in the falcon tubes, corresponding to distances between 5 and 10 mm. Alginate solution concentrations ranging from 0.8%_w_ to 3.2%_w_ were tested. The applied centrifugal speed was ranged from 1,000 to 3,000 rpm.

### 2.4 Layer-by-layer entrapment of cells in microbeads

Entrapment of *P. putida* cells in microbeads was performed using a 40 g·L^−1^ (4%_w_) alginate solution thoroughly mixed with the cell suspension at a 1:1 ratio, resulting in a 20 g·L^−1^ final alginate concentration. The cell-alginate suspension was added into the cut syringes and the bead formation was carried in 10 min cycles using a 5810 R Centrifuge with A-4-62 rotor and adaptors for four 50 mL tubes (Eppendorf, Germany) at 2,500 rpm and temperature controlled at 4 °C, with h = 5 mm. After each cycle, Falcon tubes were vortexed to prevent agglomeration of the generated beads at the bottom of the tube due to centrifugal force. Additional cell-alginate suspension was then added to the syringe and gently mixed with a pipette to avoid sedimentation of cells. Microbeads were allowed to crosslink for 20 min, after which the CaCl_2_ supernatant was discarded. Beads were rinsed twice with Milli-Q water and resuspended in Milli-Q water. To remove potential “satellites” generated during the centrifugation, the suspension was filtered using 150 µm pore size pluriStrainers (pluriSelect®, Germany). The resulting microcapsules are referred to as Alg. For additional coating, beads were covered with poly-L-lysine (PLL) *via* dip coating in a 1 mg·mL^−1^ (0.1%_w_) PLL solution for 1 h under gentle agitation using a VWR multimix rotator. Beads were then recovered, washed using Milli-Q water and resuspended in Milli-Q water until use. The resulting microcapsules are referred to as Alg-PLL.

### 2.5 System characterization and performance

#### 2.5.1 Optical microscopy

Microcapsules were imaged using a digital camera connected to an inverted microscope (Olympus IX50, Japan) in transmission mode with a bright-field illumination. Objectives with magnifications ranging from 5× to 10× were used, depending on the beads size. The focal plane was adjusted to clearly capture the contour of the capsules.

#### 2.5.2 Cryo-SEM

Empty or cell-containing alginate microcapsules (initial cell suspension OD_600_ = 35) were characterized by cryo-scanning electron microscopy (cryo-SEM). Experiments were performed using a Zeiss Crossbeam 550 Cryo-FIB-SEM equipped with a Quorum PP3010Z cryo-transfer system. Water was removed from bead suspension and beads were mounted onto an aluminium stub using carbon conductive glue. The stub was secured on the specimen holder. The sample was then immersed into liquid nitrogen and transferred into the preparation chamber under vacuum. A fractured surface of the gel was obtained by mechanically breaking the top part of the sample with a knife inside the chamber. The sample was then sublimed inside the SEM chamber, to remove residual water while minimizing structural distortions and imaging artifacts. To prevent charging, the sample was sputter-coated with platinum. Imaging was performed at accelerating voltages of 1.00, 3.00 or 5.00 kV and a working distance of 3.5–8.6 mm. Images were acquired using a secondary electron detector. Image analysis and, when applicable, colorization were performed using ImageJ 1.54p software.

#### 2.5.3 Cell escape from capsules

Cells were cultured and resuspended to reach optical densities (OD_600_) of 110, 11 and 1.1. Alg and Alg-PLL microcapsules were prepared as described above with these 3 cell optical densities. Capsules were then immersed in water at a 1:9 volume ratio (100 µL beads in 900 µL water) in Eppendorf tubes and kept at room temperature without agitation. Water was obtained from Villard de Lans (France). Details about water quality and composition can be found in Supplementary Material (Supplementary Table S1). After 6 h, 30 h and 6 days, 100 µL of the supernatant were plated onto a LB-agar plate, which were then incubated overnight at 30 °C. Colonies were then counted on each plate. CFU. mL^−1^ values were capped at 50,000 when colonies coalesced and could not be reliably enumerated as discrete units. Two Eppendorf tubes were prepared for each condition (cell concentration, Alg or Alg-PLL, days of immersion), and two LB-agar plates were prepared per tube, resulting in four data points per condition, resulting in four technical replicates. Water was plated as sterility control, yielding no colony growth. Empty alginate beads were used as negative controls, and plating of their supernatant also resulted in no colony growth.

#### 2.5.4 Capsules stability to different water salinities

Empty Alg and Alg-PLL microcapsules were characterized using optical microscopy before and after immersion for 1 week in large excess of NaCl solutions at concentrations of 0, 1, 3, 5, 7, 10, 20 g·L^−1^. Capsule swelling was quantified by measuring the average capsule diameter using ImageJ 1.54p software.

### 2.6 Viability

#### 2.6.1 Staining

Alg-PLL capsules containing *P. putida* EM42 with GFP expressing plasmid were stained with a LIVE/DEAD® BacLight™ Viability Kit for microscopy and quantitative assays (Invitrogen). Capsules preparation was achieved using an OD_600_ = 3 cell suspension to ascertain easier image analysis and correct distinction of bacterial cells. A solution of propidium iodide (PI) and SYTO® 9 stains from the LIVE/DEAD® BacLight™ Bacterial Viability Kit (1:1 ratio) was prepared. 2 μL of the SYTO® 9/PI mix was added to 1 mL of microcapsules aqueous suspension (1:5 beads to water ratio) and incubated at room temperature for 20 min in the dark before microscopy.

#### 2.6.2 Laser scanning confocal microscopy (LSCM)

200 µL of microcapsules suspensions was deposited in an 8 well µ-Slide with glass bottom (ibidi). LSCM was performed on microencapsulated bacteria with a ZEISS LSM880 (Carl ZEISS, Germany) system with a Plan-Apochromat 40×/1.4 oil immersion objective. Z-stack acquisitions, with a z-step of 1 μm, were performed on five different beads for each condition (*i.e.*, time after encapsulation). All z-stacks were performed with z-step size of 1 µm and of minimal height of 90 µm to image at least half of the beads. Frame were centered on the beads. Fluorescent images were acquired in frame mode, with a sequential collection of fluorophores, using lasers at an excitation wavelength of 488 nm and 561 nm for Live (SYTO® 9) and Dead (PI) cells respectively. Emission wavelengths were set at 521 nm and 595 nm respectively. Laser power and master gain were adjusted at 0.2/750 and 3/900 for SYTO® 9 and PI respectively, for each microcapsule in order to have a clear signal on the full stack. Pixel resolution was 512 × 512.

#### 2.6.3 Image processing

All acquired images were analysed through Fiji (ImageJ 1.54p) to quantify live or dead cells. Each channel (SYTO® 9 or PI) was processed individually using the 3D Objects Counter plugin with a threshold set between 65 and 75, allowing a clear distinction of cells over the stack. Because stacks were not of identical height and some images contained parts of surrounding beads, the ratio of live or dead cells was calculated rather than absolute cells counts, using this formula:

$$Live\;\left(or\;dead\right)\;cells\;ratio=100\times \frac{Live\;\left(or\;dead\right)\;cells}{Live\;cells+Dead\;cells}$$

### 2.7 Sensing activity

#### 2.7.1 Single-use fluorescence induction assay

The fluorescent response of encapsulated model sensor cells was evaluated in conditions of single use. Alginate (Alg) and Alginate-Poly-L-lysine (Alg-PLL) microcapsules were prepared with a PPGFP cell suspension at an optical density (OD_600_) of 35. Capsules were resuspended at a 1:5 ratio in milli-Q water with addition of 50 μg·mL^−1^ kanamycin, in order to retain relevant plasmids, and kept in a loosely capped 50 mL falcon tube at room temperature with no agitation. At different time points over a month, aliquots of capsules suspension were pipetted into black, flat bottom, 96-well microplates (ThermoScientific). In each well, 20 µL of 0, 1.1, 11, 55 mM rhamnose were added, followed by 200 µL of capsules suspension to reach 0, 0.1, 1 or 5 mM of rhamnose. All conditions were tested in triplicate. Fluorescence was measured every hour over 16 h using a Tecan Infinite® M1000 microplate reader (λ_em_ = 395 nm and λ_ex_ = 509 nm). When fluorescent increase is displayed, the endpoint fluorescence after 16 h was considered, subtracted from the initial residual fluorescence before induction. This experiment was carried out 3 different times to account for block effects when working with live cells. Sensing activity of planktonic cells was also evaluated in black, clear bottom, 24-well microplates (ibidi®). Wells were filled with 900 µL 50 μg mL^−1^ kanamycin aqueous solution, 100 µL PPGFP cells from an overnight culture in LB and 19 µL of rhamnose 275 mM to reach a final concentration of 5 mM rhamnose. Fluorescence was measured every 10 min and automatic agitation of the plate was carried between measurement (orbital, 2 mm amplitude).

#### 2.7.2 Effect of nutrients on sensing activity

To evaluate the influence of the presence of nutrients in the storage broth on fluorescent activity, the experiment was also conducted by resuspending capsules in a self-prepared medium (0.75% trehalose, 1% peptone, 0.5% yeast extract, 50 μg·mL^−1^ kanamycin) and capsules were thoroughly rinsed before use to avoid fluorescent contribution from the medium. Common LB broth could not be used since its NaCl content results in swelling and disrupting of prepared capsules.

#### 2.7.3 Effect of storage temperature on sensing activity

Evaluation of the impact of encapsulated cells storage temperature, freshly prepared capsules containing PPGFP cells were stored at room temperature (RT) fluctuating between 15 °C and 20 °C, 4 °C in a 50 μg·mL^−1^ kanamycin-containing water solution or at −20 °C in a 1:1 (v/v) mixture of 100% glycerol and kanamycin solution, in aliquots. Samples stored at −20 °C were thawed for 4 h before inducing with 5 mM rhamnose and measuring fluorescent as described above.

#### 2.7.4 Effect of cell load

Similarly, to evaluate the impact of different cell load on sensing performances, PPGFP cell suspensions at different OD_600_ were encapsulated as described and fluorescent activity was monitored overnight upon induction with 5 mM of rhamnose, following the same microplate assay protocol.

#### 2.7.5 Reusability of encapsulated cells for sensing purposes

To investigate sensor reusability and overcome the slow degradation of GFP, a PPGFP-SsrA cell suspension (OD_600_ = 35) was encapsulated in Alg-PLL. The SsrA degradation tag allowed previously produced GFP to be tagged reducing background fluorescence prior to each sensing cycle. Capsules were incubated in a 50 μg mL^−1^ kanamycin aqueous solution at a 1:5 (v/v) capsules-to-water ratio in black, flat bottom, 96-well plates (200 µL per well). GFP production was induced by adding 20 µL of 55 mM rhamnose to each well, achieving a concentration of 5 mM. Plates were incubated and monitored overnight at room temperature, using Tecan Infinite® M1000 microplate reader (λ_em_ = 395 nm and λ_ex_ = 509 nm). After 16 h of monitoring, capsules were thoroughly rinsed with milli-Q water thanks to cell strainers (100 µm pore size pluriStrainers, Pluriselect) and resuspended in milli-Q water supplemented with kanamycin (50 μg mL^−1^). Capsules were kept for 8 h in a falcon tube covered with a BREATHseal™ sealer (Greiner Bio-One Intl, Austria) under static conditions at room temperature. For reuse, capsules were resuspended at a 1:5 ratio in fresh self-prepared media, and the induction protocol was repeated. A control “single use” condition was mimicked by discarding beads and using some from the stock (encapsulated on day 1). Nine replicates were performed.

### 2.8 Diffusion

#### 2.8.1 Comsol modelling

COMSOL simulation of static diffusion in a chamber was used to predict the time-dependent gradient of inbound pollutants in the alginate bead. Although microcapsules morphology, alginate diffusion coefficient and cells repartition are assumed to be uniform in all directions, a three-dimensional model was initially implemented to account for the distribution of water sample in the chamber, around the capsules. The *Transport of diluted species in porous media* physics model was applied to a sphere (ø 180 µm) centered in a cylinder (h = 200 μm, ø 300 µm) representing the chamber. The 3D model was reduced to a 2D model by exploiting symmetry conditions to decrease the computational cost. Diffusion coefficient of studied pollutants were based on literature data and initial concentration of PECs was based on maximum value allowed in the European Union (Supplementary Table S2). In the specific case of L-rhamnose, a proxy diffusion coefficient (D_aq_ = 7 × 10^10^ m^2^/s) was used, due to its similar molecular size and hydrodynamic radius to known data from other monosaccharides (Mogi et al., 2007) and starting concentration was set at 5 mM.

The corresponding coefficients for the 2%_w_ calcium alginate gel (20 g·L^−1^) were determined by multiplying the water coefficient by a diffusion retardation coefficient of 0.97^4/3^. Indeed, effective diffusion coefficient can be defined as

$$D_{gel}=\frac{\varepsilon }{\tau }D_{aq}$$

With Dgel_Φ0_ the effective diffusion coefficient in the porous media (cell free alginate hydrogel), D the diffusion coefficient in water, ε the porosity of the alginate hydrogel and τ the tortuosity. A common approach is resorting to Millington and Quirk model (Millington and Quirk, 1961), where τ = ε^−1/3^, resulting in

$$Dgel_{\Phi 0}=\varepsilon^{4/3}D_{aq}$$

Based on literature, ε for Ca-alginate 2%_w_ is approximated to 97% (Pathak et al., 2010), thus we obtain a retardation coefficient of 0.97^4/3^ and a respective ratio D_eff_/D of 96%, in the range of what has been published by Axelsson and Persson for molecules with similar hydrodynamic radius, in alginate 2%_w_ (Axelsson and Persson, 1988).

When considering the influence of biomass content (Axelsson and Persson, 1988; Øyaas et al., 1995), D_eff_ was calculated as

$$Deff=D{gel}_{\Phi 0}\times \frac{1-\Phi c}{1+\frac{\Phi c}{2}}$$

With Φc the biomass volume fraction. For an initial cell suspension of OD_600_ = 35, cell volume fraction was measured to be 2.6%.

### 2.9 Data processing

All data processing was performed using R software version 2025.09.0. When relevant and because measurements were based on technical replicates, descriptive statistics (mean and standard deviation) were preferred over weak statistical comparisons. Results presented highlight reproducibility and the magnitude of the observed differences.

## 3 Results

### 3.1 System characterization and performance

Alginate capsules are typically prepared by dripping a sodium alginate solution into a CaCl_2_ bath using a syringe or similar device producing capsules diameters in the millimeter range (Moya-Ramírez et al., 2022). Upon contact ionic crosslinking leads to hydrogel formation through the “egg-box” structure coordinated by Ca^2+^ ions (Makarova et al., 2023). To obtain Ca-alginate microcapsules with diameters below 200 μm, a centrifugal method was optimized. The final parameters were set as follow: 2%_w_ sodium alginate solution, a needle with a 60 µm tip hole diameter, a 10 mm distance (h) between the needle tip and the 100 mM CaCl_2_ bath, and a rotational speed of 2,500 rpm (Supplementary Figure S2A). Under these conditions, capsules with an average diameter of 186 ± 4 µm were obtained (Supplementary Figure S2B). This centrifugal process enables the rapid production of highly monodisperse microcapsules in the micrometer range. Reducing capsule diameter provides several advantages: shorter diffusion paths of both incoming pollutants and outgoing response molecules, thereby accelerating sensing kinetics, and improved compatibility with miniaturized sensing devices. Freshly harvested cells were incorporated into alginate before centrifugal jetting. Optical microscopy confirmed homogenous cell distribution and effective entrapment within the resulting microcapsules (Supplementary Figure S2C).

Bacterial alginate microencapsulation is particularly sensitive to the chemical environment, as Ca^2+^ egg boxes crosslinks can be destabilized by sodium-containing surrounding media or buffers, disrupting the ionic cross-links, leading to bead swelling and therefore the release of encapsulated cells. Work carried out by Bassut et al. showed the contribution of compatible buffers (*e.g.,* HEPES) was not significant in the bioconversion performance of encapsulated bacterial cells stored in distilled water in the context of biocatalytic reactions (Bassut et al., 2025). Accordingly, microcapsules were kept in water in this work.

**FIGURE 1:**
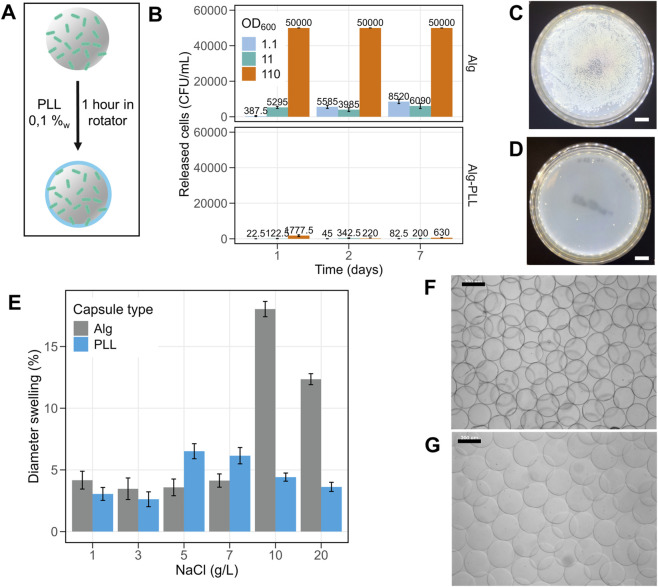
**(A)** Illustration of layer-by-layer process. Bacteria are shown in green, and the blue line represents the PLL layer. **(B)** Released CFU/mL from immersed Alg or Alg-PLL microcapsules over time (n = 4 technical replicates, showing standard deviation) for OD_600_ = 1.1, 11 and 110 of initial cell culture. The reported maximum value of 50,000 CFU/mL is arbitrary, as large colony merging prevented the identification of distinct individual colonies. Agar plates with supernatant from Alg **(C)** or Alg-PLL **(D)** capsules loaded with PPGFP OD_600_ = 110. Scale bar is 1 cm. **(E)** Diameter swelling of Alg and Alg-PLL microcapsules after 7 days of immersion in NaCl, 30 ≤ n ≤ 40 technical replicates, showing standard deviation. Optical microscopy images of Alg before **(F)** and after **(G)** 7 days immersion in 10 g·L^−1^ NaCl. Scale bars are 200 µm.

To address these challenges and to protect the cells and the capsules from environmental changes while enhancing cell containment, a layer-by-layer approach was followed (Figure 1A). In this study, a poly-L-lysine (PLL) polycationic overlayer was used, which ionically binds to the polyanionic alginate surface. PLL was selected because of its strong performance in previous studies (Moya-Ramírez et al., 2022; Bruyant and Coradin, 2007). The effectiveness of the LbL strategy was evaluated in terms of cell release and protection from environmental stress when capsules were statically immersed in water. Cell release from Alg capsules increased both with incubation time and initial cell loading (Figures 1B,C). In contrast, Alg-PLL capsules exhibited a drastically lower number of released cells of approximately two orders of magnitude (Figures 1B–D). In addition, no correlation was observed between immersion time or cell loading and the number of escaped cells, suggesting that the released cells were residual surface-associated bacteria or originated from poorly coated regions. PLL also provides protection against environmental factors, in particular the presence of salts, which is relevant when monitoring brackish or salty water. After 1 day of immersion in various concentration of sodium chloride, 2%_w_ Alg capsules showed an increasing swelling rate when exposed to 10 g·L^−1^ NaCl or higher, reaching 18% (Figures 1E–G). In contrast, PLL-coated capsules displayed constant and limited swelling, remaining below 5% in saline solutions (Figure 1E).

**FIGURE 2:**
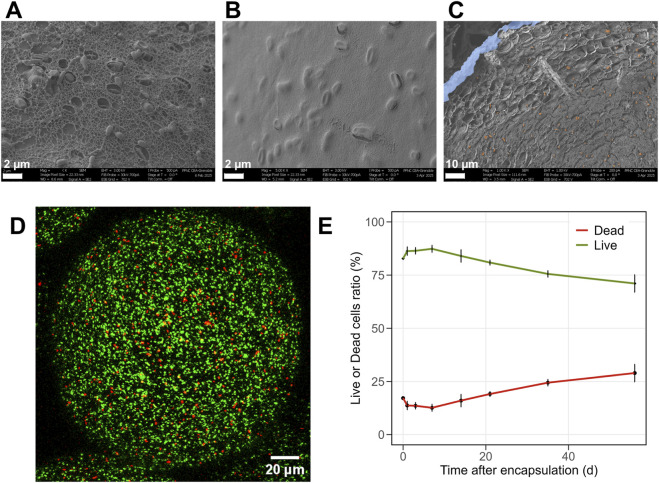
CryoSEM micrographs of Alg **(A)** and Alg-PLL **(B)** with PPGFP (initial cell culture OD_600_ = 35). **(C)** Colorized cross section of an Alg-PLL microcapsules containing bacteria, with the PLL layer shown in blue and the bacteria in orange. **(D)** Confocal microscopy image of a Live/Dead stained Alg-PLL microcapsules contaning PPGFP cells (initial cell culture OD_600_ = 3), with z-projection of maximum intensity point for each bacteria. **(E)** Live or dead cell ratio in microcapsules after encapsulation and storage at room temperature showing standard deviation (n = 5 technical replicates).

To gain a better understanding of the mechanisms underlying the reduction in cell release, cryo-SEM observations of the surface of Alg and Alg-PLL beads were carried out, revealing clear differences in morphology (Figure 2). The Alg capsule surface displays characteristic features of a Ca-alginate gel network with visible bacteria (Figure 2A). Cells appear either deposited on or embedded within the network. Some empty holes are observed, likely corresponding to former bacterial embedding sites from which cells have escaped into the environment. In contrast, the Alg-PLL surface shows a smooth and uniform surface texture, with some patterns corresponding to underlying bacterial cells (Figure 2B). These cells are well trapped between the two layers. No hole or damage are visible in the PLL layer, explaining the drastically lower number of released cells. Further cryo-SEM images of an Alg-PLL capsule cross section display clear elements of the immobilization system: the polymer network, bacterial cells and the PLL layer in the nanometer range (Figure 2C). These images show the porous structure of alginate, a homogeneous distribution of cells within the bead, and the PLL layer confining the cells and preventing their release.

### 3.2 Viability of encapsulated bacterial cells

After characterizing the structural features of capsules and physical entrapment of cells, we next examined whether these properties were compatible with the survival of the encapsulated cells by assessing cell viability. PPGFP cell-containing microcapsules were sampled at 0, 1, 2, 4, 7, 14, 21, 35, and 45 days after encapsulation. Bacterial cells were subsequently stained using a Live/Dead assay kit to distinguish live and dead cells. Figure 2D shows a z-projection of acquired stack. Confocal z-stack images reveal spherical capsules with almost no bacteria outside the beads. Image analysis shows a slight decline in the proportion of live cells overtime (Figure 2E), highlighting the cytocompatibility of the Ca-alginate encapsulating matrix with bacterial cells in month-long time frames. In addition, 3D reconstruction of the capsules allowed the determination of the distribution of dead cells, demonstrating no particular concentration of dead cells either at the core of the capsules sphere or at the periphery (Supplementary Video, Supplementary Figure S3). Indeed, while previously published studies have reported potential limitations in oxygen accessibility or increased environmental stress for cells located either far from the borders or at the periphery of the capsules (Sønderholm et al., 2017), such findings were not observed in our system.

Nevertheless, the Live/Dead assay provides information on membrane integrity rather than on cellular vitality or metabolic activity. Therefore, since this study is conducted in the context of biosensing device development, the sensing activity of the model bacterial sensors was further investigated.

### 3.3 Sensing activity

**FIGURE 3:**
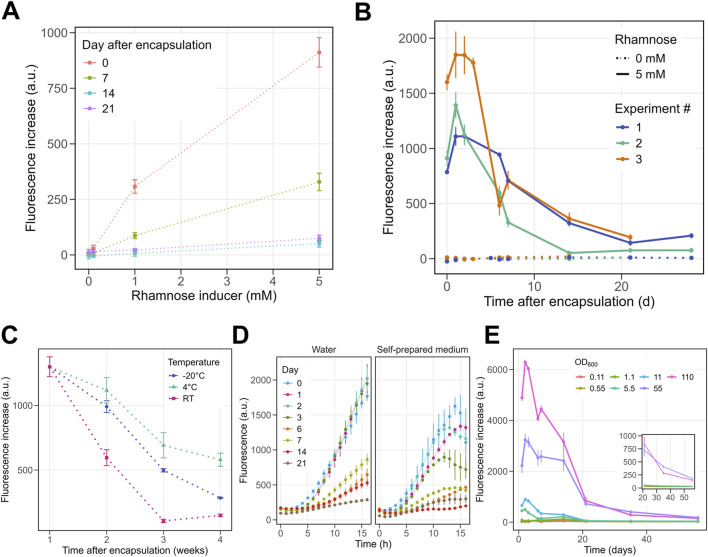
Fluorescent increase of Alg-PLL microcapsules with PPGFP **(A)** induced with different rhamnose concentrations, **(B)** over different block experiments. **(C)** Initial cell culture OD_600_ = 35 and stored at −20 °C, 4 °C, or room temperature **(D)** with different cell loads of PPGFP after storage at room temperature, **(E)** initial cell culture OD_600_ = 35 and stored in water or in nutritive medium. All panels show data obtained from 3 technical replicates, with error bars being standard deviation.

To assess the sensing response, PPGFP cells were microencapsulated and induced with rhamnose, resulting in sfGFP production as the output signal. Figure 3A shows the fluorescent response of loaded capsules to a range of rhamnose concentrations with increasing signal observed as the inducer concentration increased. Induction with 0.1 and 1 mM rhamnose resulted in fluorescence levels comparable to those of the negative control (0 mM) after 7 and 14 days respectively, indicating that the loaded capsules progressively lose sensibility over time. Among the tested rhamnose concentrations, only 5 mM triggered a detectable increase in fluorescence at 14 and 21 days. These results indicate a time-dependent increase in the limit of detection (LoD), with the biosensor no longer responding to lower inducer concentrations after prolonged encapsulation. Furthermore, when working with living organisms, it was deemed important to study the repeatability of assembled biosensing capsules by repeating the experiment with different cell batches. A similar overall behavior can be observed, with an initial fluorescence increase plummeting after a week and decreasing more slowly between 7 and 21 days, but still delivering detection above negative control (Figure 3B). Nevertheless, the three different block experiments showed varying fluorescence increases. This is one of the core challenges of biotechnologies, where inherent biological variability coupled to small experimental variations can generate visible consequences (Becker et al., 2023). Therefore, engineering biosensing cells with a “vitality” marker, that would allow to normalize produced data, both over batches and overtime, should be considered. Surprisingly, fluorescence increase was still observed for at least 28 days after encapsulation (Figure 3B) and storage in water.

Key encapsulation parameters were investigated, in particular initial cell loading in the capsules and storage conditions (nutrients supply and temperature). Storage conditions significantly influenced the fluorescent response of loaded microcapsules. The results showed optimal fluorescence when stored at 4 °C, likely due to reduced metabolic activity at lower temperatures (Figure 3C). Although the sensor is designed for use in environmental watercourses at room temperature, these findings emphasize the potential for long-term storage of unused assembled devices at 4 °C to preserve sensor performance. Regarding storage in a nutrient-containing medium before sensing, no improvement was observed with the self-prepared medium (Figure 3D). Indeed, the metabolic activity of induced encapsulated cells appeared faster, with a response occurring approximately 1 h earlier than when stored in a water solution but reaching a lower maximum, and in a less predictable time-frame (12 h on day 3 and >16 h on day 6), rendering future automated data analysis more delicate. Capsules stored in water did not seem to have reached their peak after 16 h, still delivering a stronger signal. Notably, water-stored microcapsules may contain bacterial cells in a dormant state which can be reactivated upon exposure to nutrients. Although rhamnose is not a preferred carbon source, it can act both as a default sugar for metabolic activity and as the triggering input molecule.

Optical microscopy images confirmed that increasing cell density led to progressively more opaque capsules, while their spherical shape and diameter remained unchanged (Supplementary Figure S4A–C). Fluorescence increases from microcapsules containing various quantity of bacterial cells (ranging from 0.11 to 110 in terms of initial cell suspension OD_600_) was monitored over time (Figure 3E). During the first 2 days, fluorescence increased proportionally with cell loading. Over time, the signal decreases rapidly, and no signal increase was detected for microcapsules with a lower cell load (up to 11). Hence, higher cell density provides a higher raw signal, corresponding to a higher limit of detection (LoD). Notably, for OD_600_ of 55 and 110, no significant difference in fluorescent was observed after 3 weeks, indicating that excessively high cell packing is unnecessary. For biosensing purposes, a high LoD and minimal cell leakage are critical parameters, which motivated the use of relatively high cell densities together with a relatively high alginate concentration (2%_w_) to produce a denser polymer network and tighter cell entrapment. No interference between the bacteria were observed, regardless of the number of encapsulated cells. This absence of interference could be explained either by quorum sensing phenomena or by the fact that strong entrapment within the polymer matrix inhibits intercellular interactions. BAdditionally, lower cell quantities do not appear to promote to cell division, as no signal increase was observed. Finally, after 2 months, the two most densely loaded capsules still exhibited fluorescence above the negative control, indicating that the response persisted. It is important to note that supernatants showed no detectable fluorescence, indicating that the signal is cell-associated and not due to GFP release after cell lysis and long turnover. These data indicate that a viable, inducible subpopulation persists within the Alg-PLL matrix and can re-activate GFP expression upon induction.

These results suggest that bacterial cells enter an encapsulation-induced dormancy. Indeed, encapsulation creates a protective environment that allows long-term viability and inducible gene expression under a diffusion-limited, low-nutrient and low-oxygen microenvironment. Results show that cells remain active for at least 2 months without addition of nutrients, indicating that a subpopulation retained metabolic sensing abilities. In addition, Live/Dead staining revealed that 75% of cells were still “viable” (non-damaged membrane). These results are consistent with findings from literature, stating encapsulation significantly slows down cell death (Yeung et al., 2016; Zeashan et al., 2020; Souza-Alonso et al., 2021).

First, this can be explained by the physical and chemical protection brought by the alginate-PLL matrix. Indeed, it shields its content from desiccation, mechanical stress, oxidative damage (Tønnesen and Karlsen, 2002). Alginate immobilization reproduces key physicochemical features of natural bacterial biofilms. In biofilms, cells are embedded in an extracellular polymeric substance (EPS) matrix, often rich in polysaccharides such as alginate, rendering more difficult access to nutrients and oxygen in microenvironments surroundings cells. These diffusion limitations create a favorable environment for cells to fall into dormancy, viable but non-culturable (VBNCs) state or ultra-slow metabolism (Ayrapetyan et al., 2015; Oliver, 2016).

Thus, alginate encapsulation can be viewed as a biofilm-mimicking system that induces dormancy-like behavior through diffusion-limited microenvironments. These cells drastically reduce their metabolic activity and energy consumption while preserving key cellular functions. Even minimal intracellular energy reserves allow these dormant cells to mount limited transcriptional and translational responses upon exposure to an inducer, *i.e.*, rhamnose in our case (Ayrapetyan et al., 2015). This phenomenon could explain the observation of low-level fluorescence increase 2 months after encapsulation despite the absence of growth. In addition, ability to survive in a dormant state is closely linked to the ability of bacterial cells to overcome oxidative stress. Here, the studied strain *P. putida* EM42 was selected for its good resistance to oxidative stress (Martínez-García et al., 2020). Rhamnose, serving as the inducer for the model cellular sensor, also functions as a carbon source, thereby providing a dual role in this system. Its nutritive properties not only stimulate metabolic activity but also decreases stress on the microorganisms, making it a relevant stimulus for enhancing cellular responses.

Overall, the persistence of inducible fluorescence months after encapsulation underlines the benefits of using encapsulation matrixes, in particular alginate, for long-term biosensing application. Alginate-PLL encapsulation thus represents a promising strategy to maintain viable, responsive microbial sensors under minimal nutrient conditions over extended periods.

### 3.4 Reusability

**FIGURE 4:**
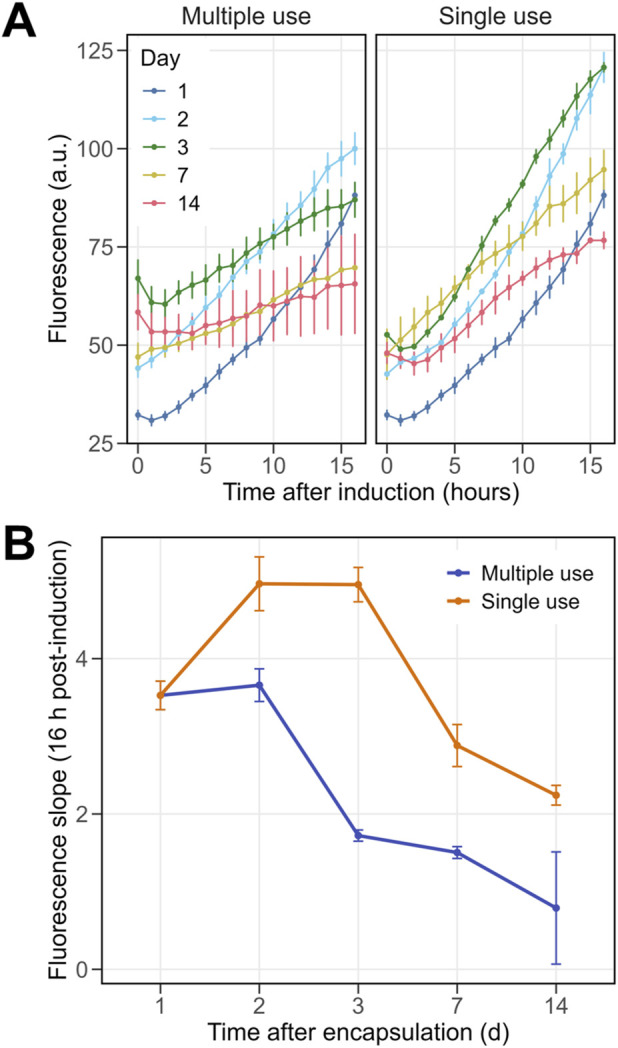
**(A)** Fluorescence response of Alg–PLL beads containing PPGFP-SsrA cells after induction with 5 mM rhamnose reusing induced beads or inducing new beads from the stock **(B)** Fluorescence slope 16 h post-induction for microcapsules used ones or reused over 2 weeks. Data were obtained from 9 technical replicates and error bars display standard deviation.

In a larger sustainability agenda, reusability of the encapsulated bacterial cells was evaluated. Because GFP is a stable protein persisting in the cell for long periods of time (Tombolini et al., 2006), a PPGFP-SsrA, was engineered to allow faster degradation of GFP after production and avoid cell saturation, thanks to a degradation tag on the GFP encoding gene. As a result, in this experiment, the overall detected GFP *via* fluorescence is unavoidably lower in intensity as GFP does not build up as much as in the PPGFP strain. This also increases the LoD of a potential biosensing system. Results obtained from 5 mM rhamnose induction of unused or reused capsules are displayed on Figure 4A. All capsules were prepared at the same time, so the results show only the effect of reusability on sensing ability. Fluorescence signal-to-noise ratio (SNR) remained above the detection threshold over four induction cycles within 1 week, although the response amplitude was reduced compared with single-use sensors. On day 7, after four induction cycles, reused sensors exhibited a fluorescence increase of 22 ± 4, compared with 47 ± 8 for single-use biosensors, corresponding to an approximately twofold stronger signal for the latter while keeping a similar SNR. Nonetheless, on day 14, following a fifth induction cycle, the SNR of reused sensors dropped below reliable detection levels (fluorescence increase of 7 ± 13), whereas single-use encapsulated bioware still showed a robust and reproducible response (28 ± 3). Focusing on slope also show a greater decrease with reused beads and their weaker signal (Figure 4B). Although signal degradation occurs with repeated use, these results demonstrate that whole-cell–based sensors can be reliably reused multiple times, supporting their potential as a more sustainable alternative to single-use disposable bioware components.

### 3.5 Diffusion across the microcapsules

**FIGURE 5:**
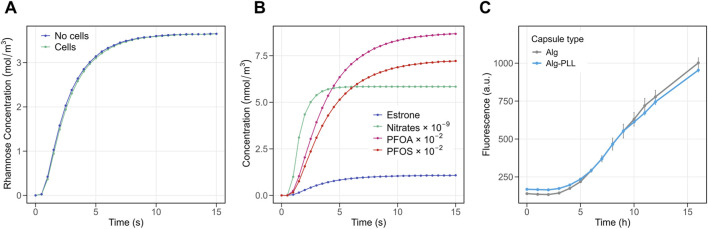
**(A)** COMSOL model of concentration of incoming rhamnose at the center of Alg microcapsules, with or without PPGFP cells encapsulated, after first contact between 5 mol·m^3^ (5 mM) rhamnose and Alg. **(B)** Concentration of various pollutants at the center of Alg microcapsules, with PPGFP cells. **(C)** Fluorescence of Alg and Alg-PLL (initial cell culture OD_600_ = 35) after induction with 5 mM rhamnose (n = 3 technical replicates, showing standard deviation).

Previous studies have shown the increase in response time of encapsulated sensors, especially when working with an additional layer deposited by LbL (Moya-Ramírez et al., 2022). To ensure that cell immobilization would not cause a dramatic decrease in sensor reactivity, we decided to model the diffusion of pollutants (*i.e.*, estrone, nitrates, PFOA, PFOS), inside a microcapsule with or without cells. Diffusion of rhamnose is shown as a representative example (Figure 5A). Data obtained by Comsol simulation show that the necessary time for targeted analytes to reach the center of the capsules is less than 15 s (Figure 5B). These values are insignificant compared to the overall “kinetics” of the system, considering that bacterial metabolism takes at least 3 hours before producing an initial fluorescence increase (Figure 5C).

Although the influence of the PLL layer on diffusion could not be modelled due to lack of data on its porosity, an experimental evaluation was carried out by comparing response times of Alg and Alg-PLL capsules after rhamnose induction, revealing no distinguishable difference (Figure 5C). For such, it is worth discussing the influence of the absence of active oxygenation in the system, which most likely slows down the bacterial response time, as planktonic cells under agitation show an initial fluorescent response 20 min after induction with 5 mM rhamnose (Supplementary Figure S5). Therefore, the response time of the biosensor is not limited by diffusion but rather relies on the metabolic response of entrapped cells under static conditions. Consequently, it is important to design a chamber that allows correct O_2_ flow in order to avoid delaying the fluorescent output from bacterial cells (Cristea et al., 2020).

## 4 Conclusion

This work demonstrates that LbL encapsulation of genetically engineered bacterial cells within Alg-PLL microcapsules enables the development of a robust, synthetic biology-driven sensing system. The encapsulation strategy provided effective cell confinement, extended viability, and long-term inducible activity, validating its suitability for integration into bimodal optical–electrochemical biosensors. The ability of encapsulated modified *P. putida* cells to remain responsive after extended storage under nutrient-depleted conditions highlights the potential of this approach for field-deployable, low-maintenance sensing platforms.

Future work should focus on the physical integration of encapsulated cells with transducing elements to achieve a fully integrated, interference-free biosensing system. Replacing the model rhamnose-induction system with analyte-specific regulatory circuits would enable direct detection of relevant environmental pollutants. Similarly, substituting the GFP reporter with molecules exhibiting dual optical and electrochemical signatures (e.g., pyoverdine) could enhance the sensing versatility of the platform. Multiplexing could be approached at two levels: either through advanced synthetic biology, by engineering a single *P. putida* strain capable of processing multiple input signals and generating distinct output responses, or at the material–device interface, by immobilizing and arranging microcapsules containing different engineered strains, each dedicated to a specific analyte, onto the transducer surface, in a “puzzle-like” configuration. Altogether, these results establish a foundational step toward next-generation living biosensors, where synthetic biology and materials engineering converge to create selective and sustainable monitoring tools for environmental applications.

## Data Availability

The raw data supporting the conclusions of this article will be made available by the authors, without undue reservation.
