# Supplementary material for: Microencapsulation of engineered bacteria towards whole-cell-based environmental biosensing
Source: Front Bioeng Biotechnol. 2026 Jun 30;14:1851287. doi: 10.3389/fbioe.2026.1851287 (PMC13365030; doi:10.3389/fbioe.2026.1851287)
Supplement: Supplementary file 1 [file Table1.docx]

Supplementary Material

# Supplementary Table

**Table S1.** Analysis of water obtained in Villard-de-Lans, France and used for cell release study from immersed microcapsules. (“Analyses of sanitary controls for waters intended for human consumption in Villard de Lans,” n.d.)

| Parameters | Value |
| --- | --- |
| Enterococci /100ml-MS | <1 n/(100mL) |
| Sulfite-reducing bacteria and spores | <1 n/(100mL) |
| Aerobic bacteria revivifiable at 22°-68h | <1 n/mL |
| Escherichia coli /100ml - MF | <1 n/(100mL) |
| Water temperature | 8.0 °C |
| Free chlorine | <0.05 mg(Cl_2_)/L |
| Total chlorine | <0.05 mg(Cl_2_)/L |
| pH | 7.70 pH unit |
| Sulfates | 0.90 mg/L |
| Chlorides | 0.70 mg/L |
| Conductivity at 25°C | 388 µS/cm |
| Ammonium (as NH_4_) | <0.01 mg/L |
| Nitrites (as NO_2_) | <0.01 mg/L |
| Nitrates/50 + Nitrites/3 | 0.07 mg/L |
| Nitrates (as NO_3_) | 3.69 mg/L |

**Table S2.** Studied pollutants diffusion coefficients in water (D_aq_), empty alginate (D_gel_) and alginate filled with bacterial cells (D_gel_Φ0_) and their initial concentrations considered in the environment. (Land et al. 2006; Kreft et al. 2001; Prasad et al. 2021; Nghiem and Schäfer 2002; Guo et al. 2017; Pereira et al. 2014)

| Studied pollutants | D_aq_ (× 10^-10^ m^2^.s^-1^) in water 25°C | D_eff___alg_ (× 10^-10^ m^2^.s^-1^) | D_gel_Φ0_ (× 10^-10^ m^2^.s^-1^) | Initial concentration (Directive (EU) 2020/2184 of the European Parliament and of the Council of 16 December 2020 on the Quality of Water Intended for Human Consumption 2020; Carere et al. 2016) | |
| --- | --- | --- | --- | --- | --- |
|  |  |  |  | mg.L^-1^ | mol.m^-3^ |
| Nitrate (NO_3_^-^) | 17 | 16.3 | 15.7 | 50 | 0.8 |
| Nitrite (NO_2_^-^) | 17 | 16.3 | 15.7 | 0.5 | 0.01 |
| Estrone (E1) | 5.4 | 5.18 | 4.99 | 0.4 × 10^-6^ | 1.48 × 10^-9^ |
| PFOA | 4.9 | 4.70 | 4.53 | 0.5 × 10^-3^ | 1.2 × 10^-6^ |
| PFOS | 4.7 | 4.51 | 4.35 | 0.5 × 10^-3^ | 1 × 10^-6^ |
| L-rhamnose | 7 | 6.72 | 6.36 | 820.8 | 5 |

# Additional references.

“Analyses of sanitary controls for waters intended for human consumption in Villard de Lans.” n.d. Accessed May 5, 2026. https://orobnat.sante.gouv.fr/orobnat/rechercherResultatQualite.do.

Carere, Mario, Luca Lucentini, Ines Lacchetti, and Robert Pasanen-Kase. 2016. “E1, E2 and EE2 in Surface and Wastewaters: An International Project under the European Water Framework Directive.” June 19, 6.

Directive (EU) 2020/2184 of the European Parliament and of the Council of 16 December 2020 on the Quality of Water Intended for Human Consumption, European Parliament and Council of the European Union, Directive (EU) 2020/2184 (2020). http://data.europa.eu/eli/dir/2020/2184/oj/eng.

Guo, Wei, Kersten Van Langenhove, Michael S. Denison, Willy Baeyens, Marc Elskens, and Yue Gao. 2017. “Estrogenic Activity Measurements in Water Using Diffusive Gradients in Thin-Film Coupled with an Estrogen Bioassay.” *Analytical Chemistry* 89 (24): 13357–64. https://doi.org/10.1021/acs.analchem.7b03537.

Kreft, Jan-Ulrich, Cristian Picioreanu, Mark C. M. van Loosdrecht, and Julian W. T. Wimpenny. 2001. “Individual-Based Modelling of Biofilms.” *Microbiology* 147 (11): 2897–912. https://doi.org/10.1099/00221287-147-11-2897.

Land, Laura M., Ping Li, and Paul M. Bummer. 2006. “Mass Transport Properties of Progesterone and Estradiol in Model Microemulsion Formulations.” *Pharmaceutical Research* 23 (10): 2482–90. https://doi.org/10.1007/s11095-006-9014-5.

Nghiem, L. D., and A. I. Schäfer. 2002. “Adsorption and Transport of Trace Contaminant Estrone in NF/RO Membranes.” *Environmental Engineering Science* 19 (6): 441–51. https://doi.org/10.1089/109287502320963427.

Pereira, Luís A. M., Luís F. G. Martins, José R. Ascenso, Pedro Morgado, João P. Prates Ramalho, and Eduardo J. M. Filipe. 2014. “Diffusion Coefficients of Fluorinated Surfactants in Water: Experimental Results and Prediction by Computer Simulation.” *Journal of Chemical & Engineering Data* 59 (10): 3151–59. https://doi.org/10.1021/je500211w.

Prasad, Alisha, Sushant P. Sahu, Sara Karoline Figueiredo Stofela, et al. 2021. “Printed Electrode for Measuring Phosphate in Environmental Water.” *ACS Omega* 6 (17): 11297–306. https://doi.org/10.1021/acsomega.1c00132.
