# Supplementary material for: Microencapsulation of engineered bacteria towards whole-cell-based environmental biosensing
Source: Front Bioeng Biotechnol. 2026 Jun 30;14:1851287. doi: 10.3389/fbioe.2026.1851287 (PMC13365030; doi:10.3389/fbioe.2026.1851287)
Supplement: Supplementary file 3 [file DataSheet1.docx]

Supplementary Material

# Supplementary Figures

**
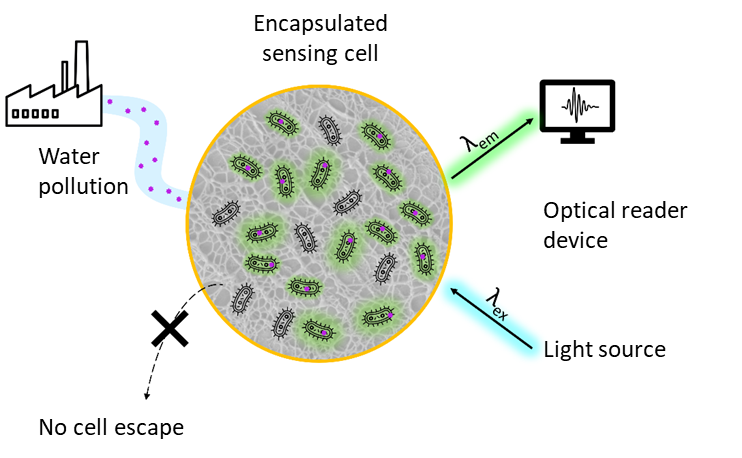
**

**Figure S1.** Graphical diagram of the whole-cell based optical sensing principle for pollutants showing the encapsulation strategy.


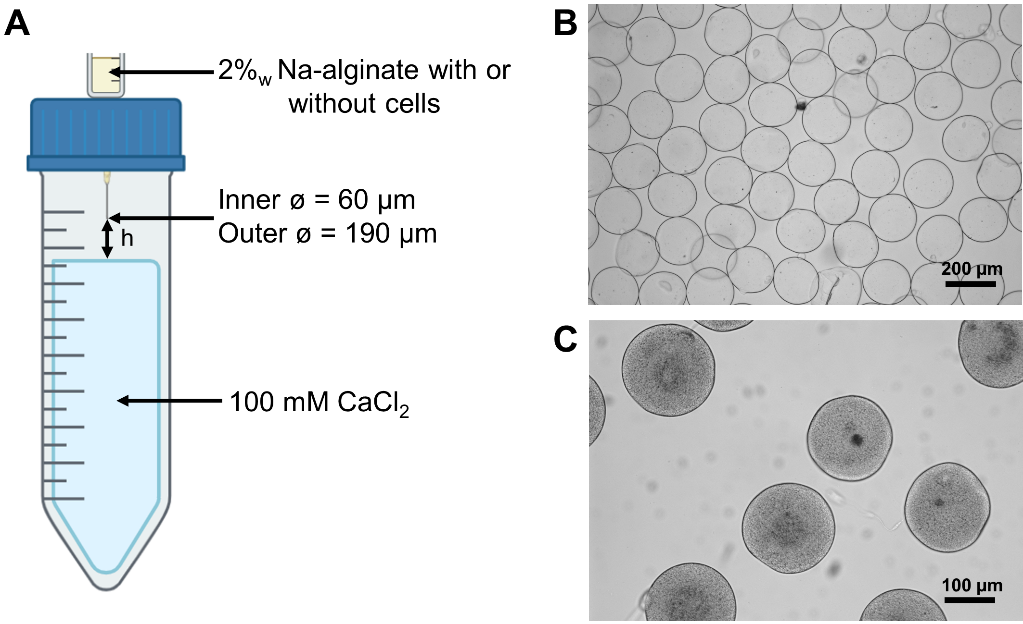


**Figure S2.** Device construction for centrifugal jetting of alginate microcapsules (**A**). Optical microscopy images of alginate microcapsules without (**B**) or with (**C**) PPGFP cells.

**Figure S3.** Confocal microscopy z-stack video reconstruction of a Live/Dead stained Alg-PLL microcapsule contaning PPGFP cells (initial cell culture OD_600_ = 3)


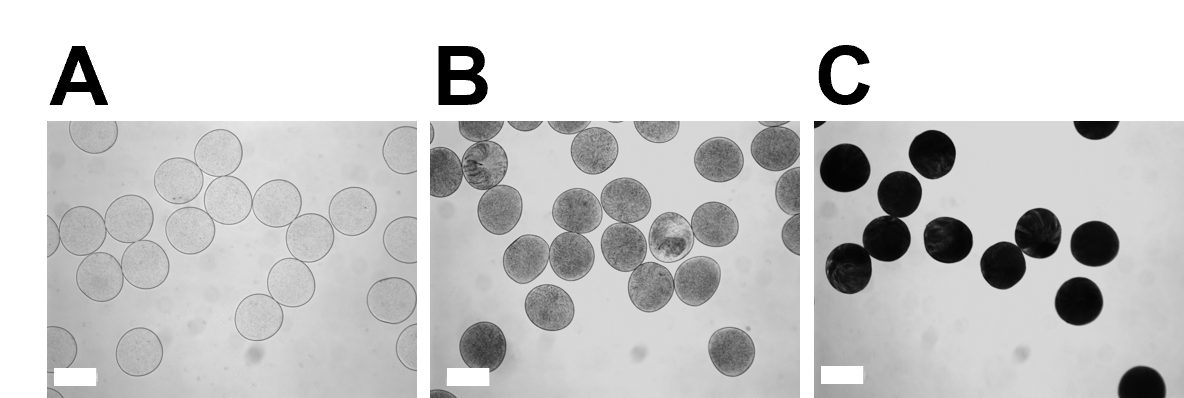


**Figure S4.** Optical microscopy images of Alg-PLL loaded with PPGFP of initial cell culture OD_600_ = 1.1 (**A**), 11 (**B**), 110 (**C**). Scale bars represent 200 µm.


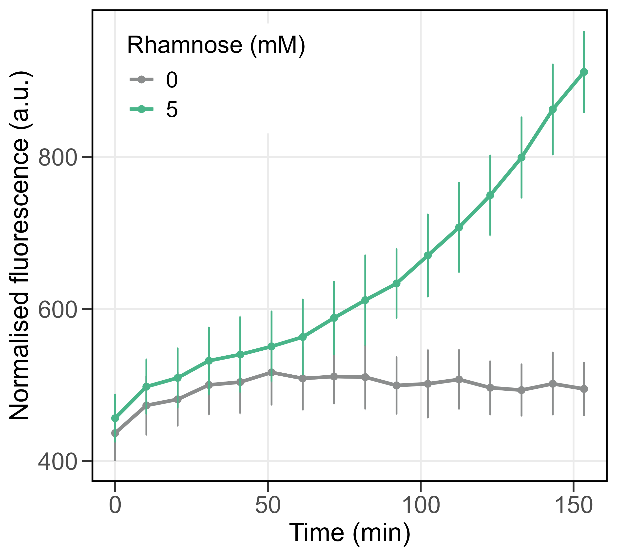


**Figure S5.** Normalized fluorescence of PPGFP planktonic cells in water after 0 or 5 mM rhamnose induction, under agitation. n=5 technical replicates, error bars represent standard deviation.
